# Case Report: Sequential treatment of growth hormone deficiency and central precocious puberty in a girl with *EP300*-mutated Rubinstein-Taybi syndrome

**DOI:** 10.3389/fped.2026.1884354

**Published:** 2026-07-29

**Authors:** Haihua Dai, Dongmei Gan

**Affiliations:** 1 School of Medicine, Ningbo University, Ningbo, China; 2 Department of Pediatrics, The Affiliated Women and Children’s Hospital of Ningbo University, Ningbo, China

**Keywords:** central precocious puberty, *EP300* mutation, growth hormone deficiency, Rubinstein-Taybi syndrome, sequential therapy

## Abstract

**Background:**

Rubinstein-Taybi syndrome (RTS) is a rare autosomal dominant disorder defined by intellectual disability, short stature, broad thumbs and halluces, and distinct craniofacial features. The genetic etiology of RTS is primarily attributed to point mutations or deletions in the CREB-binding protein gene (*CREBBP*) or the E1A-binding protein gene (*EP300*). Endocrine abnormalities in RTS remain underreported.

**Case presentation:**

A 7-year-9-month-old girl with characteristic facial features of RTS presented with growth retardation [height −2.09 standard deviation score (SDS)] and premature thelarche. Genetic and endocrinological assessments confirmed *EP300*-mutated RTS complicated by growth hormone deficiency (GHD) and central precocious puberty (CPP). Sequential therapy was initiated: recombinant human growth hormone (rhGH) monotherapy, followed by combination therapy with rhGH and gonadotropin-releasing hormone analog (GnRHa), and ultimately transitioned to GnRHa monotherapy. Her height SDS reached a peak of −0.19 during rhGH therapy. Following rhGH withdrawal, her height SDS dropped to −0.48 at 10 years and 7 months of age. Her body mass index (BMI) increased from 13.88 to 20.68 kg/m^2^ throughout the clinical course.

**Conclusions:**

In children with *EP300*-mutated RTS and short stature, evaluation for GHD via growth hormone stimulation testing should be considered, as rhGH and GnRHa therapy is effective. However, in children with RTS who have not yet reached final adult height and retain residual growth potential, premature rhGH withdrawal may compromise acquired height benefits and exacerbate weight gain. Regular monitoring of growth velocity, pubertal progression, and metabolic parameters is essential to optimize long-term outcomes.

## 1 Introduction

Rubinstein-Taybi syndrome (RTS), also known as broad thumb-hallux syndrome (OMIM#180849), is an uncommon autosomal dominant genetic disorder characterized by intellectual disability, distinctive craniofacial features (arched bushy eyebrows, beaked nose, and high-arched palate), limb malformations, growth retardation, and delayed psychomotor development. The prevalence of RTS is similar between males and females, estimated at approximately 1 in 100,000 to 1 in 125,000 individuals (1). In 1995, Petrij et al. (2) found that, in addition to rearrangements of chromosome 16p, pathogenic variants in the CREB-binding protein (*CREBBP*) gene contribute to RTS, thereby establishing its molecular basis. The diagnosis of RTS is based on two primary modalities: a clinical feature scoring system (score ≥12) and molecular genetic confirmation, which together constitute a modern diagnostic framework integrating phenotypic and genotypic data (3). *CREBBP* and its homolog, E1A-binding protein (*EP300*), encode the transcriptional coactivators CBP and p300, respectively; pathogenic variants in either gene account for RTS. Approximately 55%–70% of clinically diagnosed cases are attributed to *CREBBP* mutations (RTS1), whereas 8%–10% result from *EP300* mutations (RTS2); the genetic cause remains unidentified in the remaining cases (4, 5).

Although postnatal growth retardation is a hallmark of RTS, several reports have documented that affected children may also exhibit growth hormone deficiency (GHD). Marzuillo et al. (6) first reported a girl with RTS harboring a *CREBBP* gene mutation who presented with GHD and pituitary hypoplasia, suggesting that *CREBBP* mutations may lead to growth hormone deficiency. Subsequently, Huh et al. (7) reported a Korean child with RTS and GHD, the patient had a normal IGF-1 level, suggesting that GHD in RTS is not always accompanied by low IGF-1. Kurosawa et al. (8) documented three female patients with RTS presenting with premature thelarche, and concluded that this isolated breast development was unlikely to be coincidental. In contrast, central precocious puberty (CPP) in RTS is exceedingly uncommon, and the clinical characteristics, diagnostic approaches, and therapeutic strategies for such cases remain poorly defined.

The co-occurrence of GHD and CPP in RTS has been rarely reported, particularly among patients with *EP300* mutations. Here, we describe the dynamic clinical management of a female patient with *EP300*-mutated RTS, who was clinically diagnosed with both GHD and CPP, enriching the limited literature on this rare phenotype. Treatment discontinuation caused marked metabolic changes, highlighting the value of ongoing multidisciplinary monitoring.

## 2 Case presentation

### 2.1 Clinical presentation

A 7-year-9-month-old female child (born on August 12, 2015) was hospitalized on June 11, 2023, presenting with a chief complaint of “slow growth over the past six years and breast development over the last three months.” The patient was delivered by cesarean section at 36 weeks' gestation, weighing 2.0 kg (small for gestational age); birth length was not documented. She has a history of growth retardation, alongside intellectual and motor delays, with a Gesell developmental quotient of 42. She can speak simple words and recite poems, but cannot comprehend word meanings.

**Figure 1:**
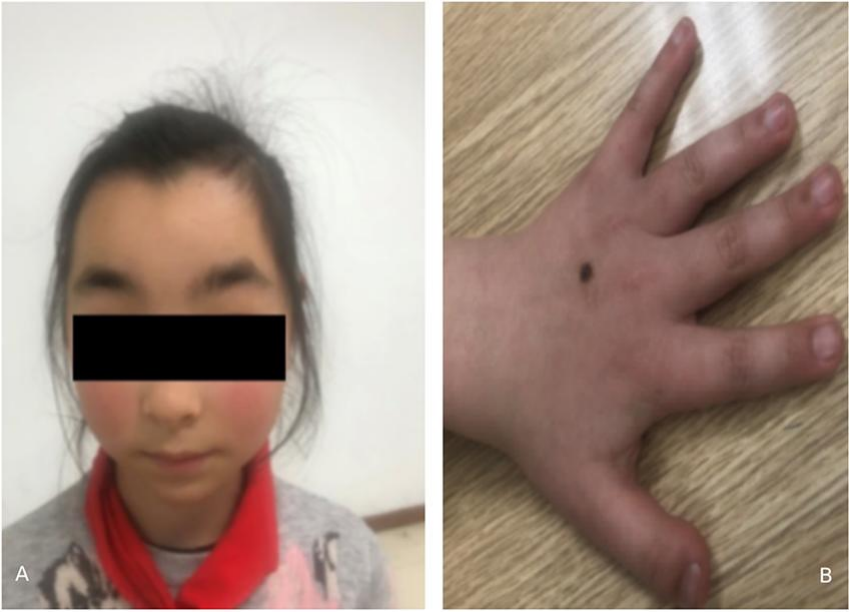
Clinical photographs of the patient. **(A)** Facial appearance. **(B)** Broad thumbs.

Physical examination revealed a height of 116 cm [−2.09 standard deviation score (SDS)] and a weight of 21 kg. Her head circumference measured 47 cm (<−2 SDS), consistent with microcephaly. The patient had arched eyebrows, ptosis, high-arched palate, hypertrichosis, and broad thumbs and halluces (See Figure 1). A comprehensive multidisciplinary evaluation revealed no significant respiratory, gastrointestinal, visual, or auditory abnormalities. There is no family history of genetic disorders, and parental pubertal development was unremarkable. Her father's height is 177 cm and her mother's is 161 cm, yielding a mid-parental genetic target height of 162.5 cm.

### 2.2 Diagnostic assessment

Laboratory evaluations revealed baseline luteinizing hormone (LH) 0.92 mIU/mL, follicle-stimulating hormone (FSH) 6.69 mIU/mL, and estradiol (E_2_) 48.00 pg/mL. Following GnRH stimulation, peak LH and FSH levels reached 18.22 and 23.19 mIU/mL, respectively. Combined evidence from hormone testing, breast development, and a bone age (BA) of 8.5 years, determined using the Greulich-Pyle atlas on a left wrist radiograph, confirmed the diagnosis of CPP. Thyroid and adrenal axes were intact: thyroid-stimulating hormone 2.9 mIU/L, free thyroxine 0.76 ng/dL; 8:00 cortisol 8.37 μg/dL with adrenocorticotropic hormone 24.25 pg/mL, and 16:00 cortisol 8.86 μg/dL with adrenocorticotropic hormone 24.08 pg/mL.

Magnetic resonance imaging revealed a pituitary height of 2.5 mm, with no other structural abnormalities. Thyroid, adrenal, renal, and abdominal ultrasonography were structurally unremarkable. The baseline insulin-like growth factor-1 (IGF-1) level was 233 ng/mL. A single combined arginine-clonidine GH stimulation assay yielded a subnormal peak of 8.78 ng/mL. Collectively, these objective findings confirmed a dual diagnosis of GHD and CPP.

### 2.3 Genetic analysis

The patient's karyotype was normal (46,XX). The diagnosis of RTS was established by detecting a *de novo* heterozygous pathogenic deletion in *EP300*. Comprehensive whole-exome sequencing and copy number variation analysis, encompassing approximately 20,000 protein-coding genes, revealed a 636-bp deletion on chromosome 22q13.2 (GRCh38/hg38, chr22:41117186–41117821). Sanger sequencing confirmed that neither parent carried this variant. Per the American College of Medical Genetics and Genomics guidelines, this variant was classified as pathogenic based on criteria PVS1, PM2_Supporting, and PP4.

### 2.4 Therapeutic intervention and follow-up

As her initial bone age advancement was relatively mild, we prioritized close pubertal monitoring over immediate GnRHa therapy. At 7 years and 11 months of age, her height was 117 cm (−2.04 SDS) and weight 19 kg. She started rhGH therapy at an initial dose of 0.13 IU/kg/day, administered via daily subcutaneous injection before bedtime. The dosage was subsequently adjusted based on growth velocity and IGF-1 levels.

At 9 years and 2 months of age, her height was 134 cm (−0.19 SDS) and weight 28 kg. She was at Tanner stage B3, with a BA of 10.5 years. Pelvic ultrasound showed uterine (25 mm × 16 mm × 24 mm) and ovary (right: 29 mm × 13 mm × 17 mm; left 28 mm × 18 mm × 24 mm) enlargement. Serum levels of LH, FSH, and E_2_ were 3.24 mIU/mL, 6.19 mIU/mL, and 49.64 pg/mL, respectively. The patient exhibited marked advancement of bone age and rapid progression of pubertal development. Based on these clinical and biochemical findings, her condition was formally classified as rapidly progressive central precocious puberty. After comprehensive shared decision-making with the patient's family, combination therapy with rhGH and GnRHa was initiated. Leuprolide acetate microspheres (3.75 mg) were administered via intramuscular injection every 4 weeks.

**Figure 2:**
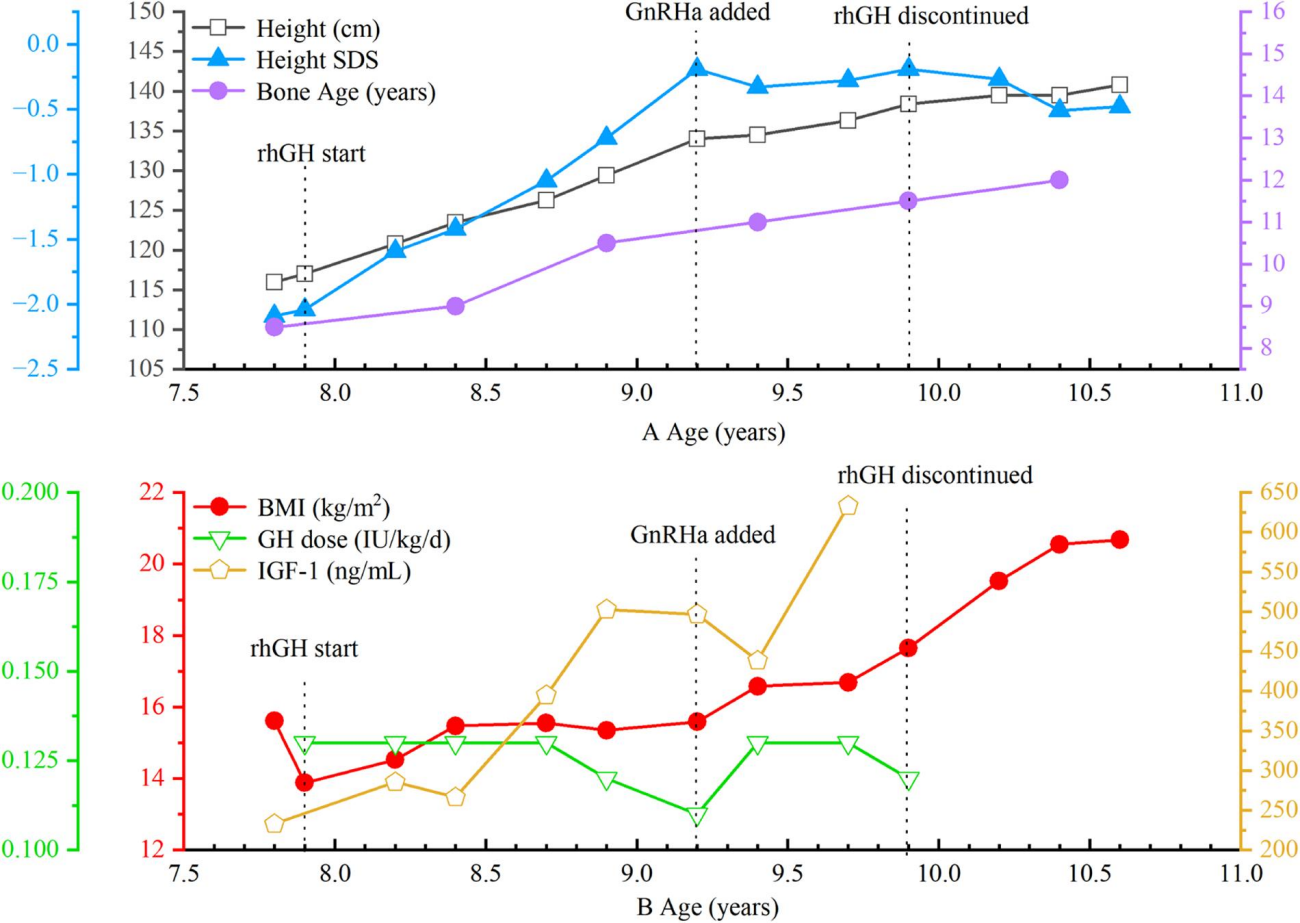
Trends in growth and metabolic parameters during treatment. **(A)** Auxological changes over time (height, height SDS, and bone age). **(B)** Corresponding changes in GH dose, IGF-1, and BMI.

Growth hormone therapy was discontinued after 2 years, when the patient was 9 years and 11 months old, with a height of 138.4 cm (−0.19 SDS) and weight 33.8 kg. This decision was driven by the family's preference for a target height of 150 cm, which they considered a reasonable compromise between an acceptable adult height and the treatment burden given her specific syndromic and neurocognitive profile. Over the treatment period, she gained a total of 21.4 cm in height: 12.4 cm during the first year and 9.0 cm in the second. Despite an observed deceleration in height velocity post-rhGH cessation, the family declined the clinical recommendation to reinitiate therapy. Vaginal bleeding occurred following the first GnRHa injection. Breast development gradually regressed to Tanner stage B2, while pubic hair remained at PH2. Serum sex hormone levels declined from baseline, whereas IGF-1 levels increased (see Figure 2 and Tables 1, 2). Thyroid, liver and kidney function remained within normal limits throughout treatment.

**Table 1:** The detailed chronological timeline of this patient's clinical management.

Age	Clinical event/intervention	Key findings/outcomes
7 years 9 months	Auxological assessment, endocrine stimulation testing, and genetic analysis.	Auxology: Height SDS −2.09; Bone Age 8.5 years.
		Endocrine: Basal E_2_ 48.00 pg/mL; GnRH-stimulated peak LH 18.22 mIU/mL and FSH 23.19 mIU/mL; stimulated peak GH 8.78 ng/mL.
		Genetics: Pathogenic deletion in the *EP300* gene.
		Outcome: Confirmed diagnosis of Rubinstein-Taybi syndrome coexisting with GHD and CPP.
7 years 11 months	Initiation of rhGH therapy.	Height SDS −2.04
9 years 2 months	Addition of GnRHa to the therapeutic regimen.	Auxology: Height SDS −0.19 (marked catch-up growth); recent Bone Age 10.5 years.
		Endocrine: Basal LH 3.24 mIU/mL, FSH 6.19 mIU/mL, and E_2_ 49.64 pg/mL; enlarged uterus and ovaries.
9 years 11 months	Discontinuation of rhGH therapy.	Auxology: Height SDS −0.19; Bone Age 11.5 years.
		Decreased sex hormone levels; pubertal progression stabilized.
10 years 7 months	Latest clinical follow-up/suggest long-term clinical monitoring	Height SDS −0.48; BMI 20.68 kg/m^2^

**Table 2:** Changes in hormone levels before and after GnRHa initiation.

Time point	LH (mIU/mL)	FSH (mIU/mL)	E_2_ (pg/mL)
Baseline (pre-GnRHa)^a^	3.24	6.19	49.64
3 months	2.41	2.32	<15^b^
6 months	1.61	1.63	<15^b^
12 months	0.80	2.09	17.55

a The patient had received rhGH monotherapy for 15 months before GnRHa initiation.

b Below the lower detection limit of the assay (<15 pg/mL).

## 3 Discussion

This case report describes a child with Rubinstein-Taybi syndrome attributable to a *de novo* heterozygous pathogenic deletion in *EP300*, who presented with GHD and CPP. Sequential administration of rhGH followed by GnRHa improved linear growth and delayed skeletal maturation. No severe adverse events were observed during active treatment. Rubinstein-Taybi syndrome was first described in 1963 (9). More than 99% of RTS cases arise from *de novo* mutations, and the recurrence risk for siblings is only 0.5%–1%, even with rare documented parental germline mosaicism (3). Prenatal course is generally unremarkable; however, affected individuals tend to develop characteristic multisystem phenotypic abnormalities gradually after birth. These encompass characteristic morphologic and neurodevelopmental anomalies, alongside cardiovascular or urogenital malformations. In addition, these patients are more susceptible to benign neoplasms (10, 11). Individuals carrying *EP300* variants tend to present with milder craniofacial phenotypes and reduced incidence of radial thumb deviation and keloid formation as opposed to *CREBBP* variant carriers. By contrast, microcephaly is far more prevalent in the *EP300* group, affecting 83%–86% of cases vs. 54% for *CREBBP* variants (12, 13). The phenotypic spectrum associated with *EP300* mutations is highly heterogeneous. RTS may resemble other chromatinopathies with short stature and facial dysmorphism, such as Cornelia de Lange, Floating-Harbor, Wiedemann-Steiner, and Kabuki syndromes (3). The patient's broad thumbs, characteristic facial features and genetically confirmed *EP300* pathogenic variant reliably distinguished RTS from these conditions.

Advanced bone age (8.5 years) and an LH peak of 18.22 mIU/mL following GnRH stimulation confirmed activation of the hypothalamic-pituitary-gonadal axis and ruled out isolated premature thelarche. At 7 years and 9 months of age, the patient was already producing endogenous estrogen, a physiological state that typically amplifies GH secretion. The patient's peak GH response to stimulation was 8.78 ng/mL, below the 10 ng/mL diagnostic cutoff for GHD established by Chinese guidelines (14), consistent with the diagnosis. Clinical evidence supports that a combined arginine-clonidine stimulation test is a reliable tool for diagnosing GHD, effectively replacing for traditional protocols while minimizing patient burden (15). Magnetic resonance imaging revealed reduced pituitary height without space-occupying lesions, providing a structural basis for GHD. Normal baseline thyroid function and morning cortisol levels, alongside an intact gonadotropic axis (evidenced by CPP), effectively excluded multiple pituitary hormone deficiency. These findings confirm true GHD rather than a false-positive result. While RTS inherently causes growth retardation, her subnormal GH stimulation test and positive response to GH therapy suggest that the patient's short stature is likely attributable to a combination of RTS-associated growth impairment and coexisting GHD.

After 2 years of rhGH therapy, her height SDS increased from −2.04 to −0.19. As exogenous growth hormone replacement, rhGH plays a pivotal role in regulating metabolism, promoting cell proliferation, and facilitating catch-up growth. The clinical efficacy and safety of rhGH replacement therapy have been widely validated, rendering it the mainstay therapeutic option for children with GHD (16, 17). Few studies have reported on growth hormone therapy among children with RTS. Marzuillo et al. (6) documented an 11-year-old girl with RTS and GHD carrying a frameshift mutation in the *CREBBP* gene; after 5 years of GH treatment, her height SDS improved from −3.5 to −2.1. Tornese et al. (18) described a 3.5-year-old girl with RTS harboring a novel *CREBBP* variant who presented with isolated GHD; following GH therapy, her height SDS increased to +0.58 at the age of 9.9 years. The 2024 international consensus on RTS states that rhGH therapy effectively improves height SDS in children with GHD (3). This clinical case provides further evidence to support this recommendation, particularly for individuals with *EP300* mutations. Given the diagnostic complexity of short stature in genetic disorders, some management guidelines for syndromic conditions emphasize GHD evaluation. As outlined in the Silver-Russell syndrome consensus, GH treatment can optimize linear growth and body composition; regular assessment of the pituitary-gonadal axis is also suggested (19). RTS children with unexplained short stature should undergo growth hormone stimulation testing regardless of genotype, as GHD may occur in association with either *CREBBP* or *EP300* mutations.

Central precocious puberty accelerates skeletal maturation, compromises final adult height, and undermines children's psychological and behavioral development (20). Xu et al. (21) proposed a risk-stratified clinical strategy recommending “watchful waiting” for isolated premature thelarche, while limiting GnRHa intervention to children with persistent pubertal progression or marked bone age acceleration. This is consistent with our clinical management. The patient showed mild bone age advancement at initial presentation; given the unpredictable course of precocious puberty, we adopted close regular follow-up. At 9 years and 2 months of age, she displayed marked acceleration in skeletal maturation and rapid pubertal progression, suggesting substantial compromise of predicted final adult height. After comprehensive discussion with the family, combination GnRHa therapy was initiated. Previous studies have shown that combined GnRHa and rhGH therapy optimize linear growth in children with short stature, with an acceptable safety profile (22, 23). During the initial phase of combination treatment, the patient experienced a temporary decline in height SDS, consistent with prior clinical observations. Evidence suggests that concurrent GnRHa and GH therapy may delay bone maturation, blunt the pubertal growth spurt, and thereby lower growth velocity (24, 25). This effect may arise from indirect suppression of the GH-IGF-1 axis, driven by GnRHa-induced inhibition of the HPG axis (26). Tanaka (27) noted that combined GH and GnRHa therapy requires a longer treatment course to compensate for the loss of the pubertal growth spurt caused by hormonal suppression. A long-term cohort study corroborates the necessity of this prolonged treatment strategy. Patients who completed a 4-year combined therapy attained a net height gain of approximately 12.0 cm relative to predicted adult height. Nearly 70% of these patients achieved their genetic target height, markedly outperforming untreated controls (28). With prolonged combination treatment, the linear growth benefit of rhGH gradually became dominant, leading to a rise in height SDS. After GnRHa initiation, the patient showed reduced sex hormone levels, slowed bone age progression, and regressive secondary sexual development, thereby preserving growth potential and optimizing final adult height.

Upon rhGH discontinuation, her predicted adult height using a modified Chinese-specific method (29) was calculated as 150.5 cm, remaining substantially below her genetic target of 162.5 cm. At that time, her bone age was 11.5 years, indicating residual growth potential. However, over the subsequent 6 months off GH therapy, she gained only 1.1 cm. The patient's height SDS gradually dropped from −0.19 to −0.48, consistent with the “GH withdrawal syndrome” described by Lampit et al. (30). In that cohort, prepubertal children with idiopathic short stature discontinued daily GH therapy either after 3 years of treatment or once their height reached the 25th percentile, with height SDS declining from −1.0 to −1.6 over 2 years. These alterations were postulated to stem from diminished responsiveness of peripheral target tissues such as growth plate chondrocytes, rather than a drop in circulating GH or IGF-1 levels (30). Sustained GnRHa treatment lowered sex hormone concentrations, whereas GH cessation removed exogenous growth stimulation. Together, these two factors may have exacerbated the patient's marked growth slowdown. In children with RTS, early discontinuation of rhGH therapy may lead to the loss of previously achieved height gains. In the long-term management of chronic conditions, shared decision-making is vital to striking a proper balance between therapeutic benefits and family preferences.

During treatment, especially after transitioning to GnRHa monotherapy, the patient's body mass index (BMI) increased markedly, from 13.88 kg/m^2^ at 7 years and 11 months to 20.68 kg/m^2^ at 10 years and 7 months. In children with CPP, studies have shown that BMI-SDS increases significantly after GnRHa treatment relative to baseline (31, 32). Estrogen facilitates lipolysis and preserves insulin sensitivity; therefore, its decline facilitates adipose accumulation. Fasting blood glucose remained within the normal range throughout rhGH therapy. Following rhGH withdrawal, the patient no longer benefited from the lipolytic action of growth hormone, further exacerbating weight gain. However, several clinical observations indicate that GnRHa-induced BMI changes are mostly transient and generally normalize after treatment cessation (33, 34). IGF-1 and IGFBP-3 concentrations are notably higher in children with CPP than in prepubertal age-matched peers (35). As an essential regulator for GH-driven skeletal growth, IGF-1 facilitates the proliferation and maturation of growth plate chondrocytes (36). The elevation in IGF-1 levels following rhGH therapy indicates a good response to treatment. At the early stage of combined GnRHa therapy, IGF-1 levels fell slightly, suggesting partial suppression of the GH-IGF-1 axis by GnRHa. During the combined therapy phase, the patient's serum total IGF-1 level peaked at 633.4 ng/mL when she was 9 years and 8 months old. Using IGF-1 reference intervals for healthy Chinese children from the PRINCE study (37) and LMS-based age interpolation, we estimated her IGF-1 SDS at +2.86. Vilmann et al. (38) reported that 25% of patients exhibited total IGF-1 exceeding +2 SDS, whereas merely 13% showed bioactive IGF-1 above this threshold, which shows that elevated total IGF-1 concentrations do not always reflect enhanced biological activity in target tissues. Marked IGF-1 elevations during GH therapy may raise long-term safety concerns, especially given historical context regarding tumor predisposition in individuals with RTS. While the 2024 international consensus concludes that for RTS patients under 40 years of age, no increased malignancy risk has been verified and routine tumor surveillance is not recommended, which supports the application of standard GH treatment protocols for this population (3). Within the current case, the patient remained free from adverse events, maintaining clinical stability without observable signs of tumorigenesis. And rhGH therapy was discontinued at 9 years and 11 months of age. Nevertheless, balancing linear growth benefits with metabolic safety remains essential. This is achieved through monitoring IGF-1 levels to guide dosage and prevent supraphysiological exposure.

## 4 Conclusions

In conclusion, for this *EP300*-related RTS patient with concomitant GHD and CPP, a dynamic management strategy effectively optimized the patient's growth trajectory. However, this study is constrained by its single-case design, and the patient's final adult height remains undetermined. The subsequent decline in height SDS and concurrent rise in BMI following rhGH withdrawal underscore the complexities of long-term syndromic management. Retrospective data collection also precluded the precise calculation of metabolic parameters, notably BMI and IGF-1 standard deviation scores. In addition, the patient's coexisting central precocious puberty created highly dynamic hormonal levels, which inherently complicated the standard interpretation of the GH stimulation test. Future clinical monitoring should include surveillance of growth velocity, serial IGF-1 levels, and metabolic profiles. Establishing collaborative patient registries is essential to accumulate robust evidence for the standardized management of complex comorbid endocrine disorders in children with RTS.

## Data Availability

The original contributions presented in the study are included in the article/Supplementary Material, further inquiries can be directed to the corresponding author.

## References

[1] Van Gils J Magdinier F Fergelot P Lacombe D. Rubinstein-Taybi syndrome: a model of epigenetic disorder. Genes. (2021) 12(7):968. 10.3390/genes12070968 34202860 PMC8303114

[2] Petrij F Giles RH Dauwerse HG Saris JJ Hennekam RC Masuno M. Rubinstein-Taybi syndrome caused by mutations in the transcriptional co-activator CBP. Nature. (1995) 376(6538):348–51. 10.1038/376348a0 7630403

[3] Lacombe D Bloch-Zupan A Bredrup C Cooper EB Houge SD García-Miñaúr S. Diagnosis and management in Rubinstein-Taybi syndrome: first international consensus statement. J Med Genet. (2024) 61(6):503–19. 10.1136/jmg-2023-109438 38471765 PMC11137475

[4] Yu PT Luk HM Lo IFM. Rubinstein-Taybi syndrome in Chinese population with four novel mutations. Am J Med Genet A. (2021) 185(1):267–73. 10.1002/ajmg.a.61922 33063428

[5] Cross E Duncan-Flavell PJ Howarth RJ Hobbs JI Thomas NS Bunyan DJ. Screening of a large Rubinstein-Taybi cohort identified many novel variants and emphasizes the importance of the CREBBP histone acetyltransferase domain. Am J Med Genet A. (2020) 182(11):2508–20. 10.1002/ajmg.a.61813 32827181

[6] Marzuillo P Grandone A Coppola R Cozzolino D Festa A Messa F. Novel cAMP binding protein-BP (CREBBP) mutation in a girl with Rubinstein-Taybi syndrome, GH deficiency, Arnold Chiari malformation and pituitary hypoplasia. BMC Med Genet. (2013) 14:28. 10.1186/1471-2350-14-28 23432975 PMC3598247

[7] Huh R Cho SY Kim J Ki CS Jin DK. Letter to the editor: a novel mutation in the CREBBP gene of a Korean girl with Rubinstein-Taybi syndrome. Ann Clin Lab Sci. (2015) 45(4):458–61. Available online at: https://webofscience.clarivate.cn/wos/alldb/full-record/WOS:000359584100015 26275701

[8] Kurosawa K Masuno M Imaizumi K Matsuo M Kuroki Y Tachibana K. Premature thelarche in Rubinstein-Taybi syndrome. Am J Med Genet. (2002) 109(1):72–3. 10.1002/ajmg.10297 11932997

[9] Rubinstein JH Taybi H. Broad thumbs and toes and facial abnormalities. A possible mental retardation syndrome. Am J Dis Child. (1963) 105:588–608. 10.1001/archpedi.1963.02080040590010 13983033

[10] Milani D Manzoni FMP Pezzani L Ajmone P Gervasini C Menni F. Rubinstein-Taybi syndrome: clinical features, genetic basis, diagnosis, and management. Ital J Pediatr. (2015) 41:4. 10.1186/s13052-015-0110-1 25599811 PMC4308897

[11] Boot MV Van Belzen MJ Overbeek LI Hijmering N Mendeville M Waisfisz Q. Benign and malignant tumors in Rubinstein-Taybi syndrome. Am J Med Genet A. (2018) 176(3):597–608. 10.1002/ajmg.a.38603 29359884 PMC5838508

[12] Cohen JL Schrier Vergano SA Mazzola S Strong A Keena B McDougall C. EP300-related Rubinstein-Taybi syndrome: highlighted rare phenotypic findings and a genotype-phenotype meta-analysis of 74 patients. Am J Med Genet A. (2020) 182(12):2926–38. 10.1002/ajmg.a.61883 33043588

[13] Van-Gils J Naudion S Toutain J Lancelot G Attié-Bitach T Blesson S. Fetal phenotype of Rubinstein-Taybi syndrome caused by CREBBP mutations. Clin Genet. (2019) 95(3):420–6. 10.1111/cge.13493 30633342

[14] Subspecialty Group of Endocrinology, Hereditary and Metabolic Diseases, the Society of Pediatrics, Chinese Medical Association. [Chinese guidelines for the diagnosis and treatment of pediatric growth hormone deficiency]. Chin J Pediatr. (2024) 62:5–11. 10.3760/cma.j.cn112140-20230914-00183 38154971

[15] Oron T Krieger A Yakobovich-Gavan M Tenenbaum A Diamant R Phillip M. Diagnosing growth hormone deficiency: can a combined arginine and clonidine stimulation test replace 2 separate tests? Endocr Pract. (2022) 28(1):36–43. 10.1016/j.eprac.2021.08.004 34418530

[16] Maghnie M Ranke MB Geffner ME Vlachopapadopoulou E Ibáñez L Carlsson M. Safety and efficacy of pediatric growth hormone therapy: results from the full KIGS cohort. J Clin Endocrinol Metab. (2022) 107(12):3287–301. 10.1210/clinem/dgac517 36102184 PMC9693805

[17] Deal CL Steelman J Vlachopapadopoulou E Stawerska R Silverman LA Phillip M. Efficacy and safety of weekly somatrogon vs daily somatropin in children with growth hormone deficiency: a phase 3 study. J Clin Endocrinol Metab. (2022) 107(7):e2717–28. 10.1210/clinem/dgac220 35405011 PMC9202717

[18] Tornese G Marzuillo P Pellegrin MC Germani C Faleschini E Zennaro F. A case of Rubinstein-Taybi syndrome associated with growth hormone deficiency in childhood. Clin Endocrinol. (2015) 83(3):437–9. 10.1111/cen.12748 25683362

[19] Wakeling EL Brioude F Lokulo-Sodipe O O'Connell SM Salem J Bliek J. Diagnosis and management of Silver-Russell syndrome: first international consensus statement. Nat Rev Endocrinol. (2017) 13(2):105–24. 10.1038/nrendo.2016.138 27585961

[20] Zevin EL Eugster EA. Central precocious puberty: a review of diagnosis, treatment, and outcomes. Lancet Child Adolesc Health. (2023) 7(12):886–96. 10.1016/S2352-4642(23)00237-7 37973253

[21] Xu Y Cui H Wang X Liu F. Management strategies for isolated premature thelarche: a risk-stratified clinical pathway favoring “watchful waiting”. Front Endocrinol. (2025) 16:1705194. 10.3389/fendo.2025.1705194 PMC12883376 41669244

[22] Bangalore Krishna K Fuqua JS Rogol AD Klein KO Popovic J Houk CP. Use of gonadotropin-releasing hormone analogs in children: update by an international consortium. Horm Res Paediatr. (2019) 91(6):357–72. 10.1159/000501336 31319416

[23] Torres-Santiago L Mauras N. Approach to the peripubertal patient with short stature. J Clin Endocrinol Metab. (2024) 109(7):e1522–33. 10.1210/clinem/dgae011 38181434

[24] Müller J Juul A Andersson AM Sehested A Skakkebaek NE. Hormonal changes during GnRH analogue therapy in children with central precocious puberty. J Pediatr Endocrinol Metab. (2000) 13(Suppl 1):739–46. 10.1515/JPEM.2000.13.S1.739 10969916

[25] Mauras N Ross J Mericq V. Management of growth disorders in puberty: GH, GnRHa, and aromatase inhibitors: a clinical review. Endocr Rev. (2023) 44(1):1–13. 10.1210/endrev/bnac014 35639981

[26] Rothman MS Wierman ME. The role of gonadotropin releasing hormone in normal and pathologic endocrine processes. Curr Opin Endocrinol Diabetes Obes. (2007) 14(4):306–10. 10.1097/MED.0b013e3281e2c9fc 17940457

[27] Tanaka T. Growth hormone therapy and puberty: the role of gonadotropin-releasing hormone analogue therapy. Curr Opin Endocrinol Diabetes Obes. (2000) 7(2):77–81. 10.1097/00060793-200004000-00006

[28] Dotremont H France A Heinrichs C Tenoutasse S Brachet C Cools M. Efficacy and safety of a 4-year combination therapy of growth hormone and gonadotropin-releasing hormone analogue in pubertal girls with short predicted adult height. Front Endocrinol. (2023) 14:1113750. 10.3389/fendo.2023.1113750 PMC10064858 37008942

[29] Liang Y Wei H Yu X Song N Luo X. [A new method for adult height prediction in girls with idiopathic central precocious puberty treated with gonadotropin releasing hormone agonist]. Zhonghua Er Ke Za Zhi. (2015) 53(11):840–4. 10.3760/cma.j.issn.0578-1310.2015.11.008 26758323

[30] Lampit M Lorber A Vilkas DL Nave T Hochberg Z. GH dependence and GH withdrawal syndrome in GH treatment of short normal children: evidence from growth and cardiac output. Eur J Endocrinol. (1998) 138(4):401–7. 10.1530/eje.0.1380401 9578507

[31] Hou L Ying Y Wu W Ye F Zhang C Luo X. The effect of GnRHa treatment on body mass index in central precocious puberty: a systematic review and meta-analysis. Horm Res Paediatr. (2024) 97(5):419–32. 10.1159/000535132 38185120 PMC11446337

[32] Zhu X Qin J Xue W Li S Zhao M Yingliang J. The effect of GnRH analog treatment on BMI in children treated for precocious puberty: a systematic review and meta-analysis. J Pediatr Endocrinol Metab. (2024) 37(4):297–308. 10.1515/jpem-2023-0416 38407229

[33] Leite AL Galo E Antunes A Robalo B Amaral D Espada F. Do GnRH agonists really increase body weight gain? Evaluation of a multicentric Portuguese cohort of patients with central precocious puberty. Front Pediatr. (2022) 10:816635. 10.3389/fped.2022.816635 35311046 PMC8931601

[34] Buyukyilmaz G Koca SB Adiguzel KT Gurbuz F Boyraz M. Body mass index evolution and ovarian function in adolescent girls who received GnRH agonist treatment for central precocious puberty or early and fast puberty. J Pediatr Endocrinol Metab. (2023) 36(11):1044–51. 10.1515/jpem-2023-0232 37735929

[35] Wu Y Zhao W. Insulin-like growth factor 1 and insulin-like growth factor-binding protein 3 in central precocious puberty: a systematic review and meta-analysis. Growth Factors. (2025) 43(2):97–105. 10.1080/08977194.2025.2521071 40600329

[36] Dixit M Poudel SB Yakar S. Effects of GH/IGF axis on bone and cartilage. Mol Cell Endocrinol. (2021) 519:111052. 10.1016/j.mce.2020.111052 33068640 PMC7736189

[37] Cao B Peng Y Song W Peng X Hu L Liu Z. Pediatric continuous reference intervals of serum insulin-like growth factor 1 levels in a healthy Chinese children population - based on PRINCE study. Endocr Pract. (2022) 28(7):696–702. 10.1016/j.eprac.2022.04.004 35430364

[38] Vilmann L Albrethsen J Petersen JH Holmboe SA Christiansen P Main KM. Bioactive IGF-I concentrations in children on GH therapy. J Clin Endocrinol Metab. (2026) 111(4):1021–30. 10.1210/clinem/dgaf566 41083160 PMC13016718

